# Anatomical Inputs From the Sensory and Value Structures to the Tail of the Rat Striatum

**DOI:** 10.3389/fnana.2018.00030

**Published:** 2018-05-03

**Authors:** Haiyan Jiang, Hyoung F. Kim

**Affiliations:** ^1^Center for Neuroscience Imaging Research, Institute for Basic Science (IBS), Suwon, South Korea; ^2^Department of Biomedical Engineering, Sungkyunkwan University (SKKU), Suwon, South Korea

**Keywords:** tail of striatum, dorsomedial striatum, sensory input, value input, habitual behavior, rostral-caudal axis

## Abstract

The caudal region of the rodent striatum, called the tail of the striatum (TS), is a relatively small area but might have a distinct function from other striatal subregions. Recent primate studies showed that this part of the striatum has a unique function in encoding long-term value memory of visual objects for habitual behavior. This function might be due to its specific connectivity. We identified inputs to the rat TS and compared those with inputs to the dorsomedial striatum (DMS) in the same animals. The TS directly received anatomical inputs from both sensory structures and value-coding regions, but the DMS did not. First, inputs from the sensory cortex and sensory thalamus to the TS were found; visual, auditory, somatosensory and gustatory cortex and thalamus projected to the TS but not to the DMS. Second, two value systems innervated the TS; dopamine and serotonin neurons in the lateral part of the substantia nigra pars compacta (SNc) and dorsal raphe nucleus projected to the TS, respectively. The DMS received inputs from the separate group of dopamine neurons in the medial part of the SNc. In addition, learning-related regions of the limbic system innervated the TS; the temporal areas and the basolateral amygdala selectively innervated the TS, but not the DMS. Our data showed that both sensory and value-processing structures innervated the TS, suggesting its plausible role in value-guided sensory-motor association for habitual behavior.

## Introduction

The striatum receives inputs from various cortical and subcortical areas, and its subregions have different functions (Selemon and Goldman-Rakic, 1985; Alexander et al., 1986; Deniau et al., 1996; Yin and Knowlton, 2006; Kim and Hikosaka, 2015). For example, the rodent dorsomedial striatum (DMS) and dorsolateral striatum (DLS) have distinct roles. The DMS guides goal-directed behavior whereas the DLS guides habitual behavior (Yin et al., 2004; Thorn et al., 2010), and the anatomical connection between these subregions suggests that striatal control of behavior shifts from DMS to DLS with habit formation (Haber et al., 2000; Keiflin and Janak, 2015). The associative and sensorimotor cortices project to the DMS and DLS, respectively, which might be critical for their distinct functions (Yin and Knowlton, 2006; Hintiryan et al., 2016). Research to date has focused on the Medial-Lateral (ML) differences in the rodent striatum. The striatum, however, is a long structure located along the rostral-caudal axis of the brain inside the telencephalon and receives inputs from various brain areas (McGeorge and Faull, 1989; Mcdonald et al., 1996; Pan et al., 2010; Hintiryan et al., 2016; Hunnicutt et al., 2016).

Recent studies showed functional differences in the rostral and caudal striatum of macaque monkey (Kim and Hikosaka, 2013, 2015). The rostral caudate nucleus (CD) guides controlled saccade, and the caudal CD guides habitual saccade. This caudal part is called tail of the CD (CDt) in primate (Selemon and Goldman-Rakic, 1985). The CDt has a critical function in long-term value memory coding for habitual saccade to valuable visual objects (visual habit). However, the rostral CD (the head of the CD, CDh) is not involved in that process (Fernandez-Ruiz et al., 2001; Kim and Hikosaka, 2013; Yamamoto et al., 2013). This selective process for visual habit suggests that the caudal and rostral basal ganglia might have different anatomical inputs. Indeed, neurons in the CDt receives input from a distinct group of dopamine neurons in caudal-lateral part of the substantia nigra pars compacta (SNc; Kim et al., 2014). These CDt-projecting dopamine neurons have a different function from CDh-projecting dopamine neurons, encoding long-term value memory (Kim et al., 2015). This selective dopamine input and its distinct function raise the question of what distinct brain-wide inputs to the caudal striatum affect its role in habitual behavior.

Visual habit can form by associating visual stimuli with their reward values (Fernandez-Ruiz et al., 2001; Kim and Hikosaka, 2013). This association is thought to strengthen the connections between sensory stimuli inputs and behavioral outputs of the reward value system (Reynolds and Wickens, 2002; Ashby et al., 2010). Based on this idea, we have a hypothesis that the brain region processing habitual behavior receives direct inputs from both sensory regions and reward-processing structures, which may be different from the inputs to the brain region processing goal-directed behavior.

To understand the role of the caudal striatum in habitual behavior, we identified the brain inputs to the caudal striatum of rat which is called the tail of the striatum (TS) and compared these with the inputs to the DMS which guides the goal-directed behavior in the same rats. Inputs from the sensory and reward systems in the cortical and subcortical structures were mainly examined. Furthermore, we investigated how the TS- and DMS-projecting neurons in each brain structure were topographically organized in the rostral-caudal axis.

## Materials and Methods

### General Procedures

Male Sprague-Dawley rats aged 9–13 weeks (Orient Bio Inc.) were used. All animal care and experimental procedures were approved by the Institutional Animal Care and Use Committee (IACUC) of Sungkyunkwan University (SKKU).

### General Surgery Procedure

Rats were initially anesthetized with 5% isoflurane in oxygen for 3 min. After deep anesthetization, the heads were fixed to a stereotaxic frame (David Kopf Instruments) for incision and injection. The percentage of isoflurane was gradually decreased for the incision (3%) and injection (2%). After tracer injection, the injection needle and injection tower were removed, and the incision was sutured with surgical silk. Animals were then returned to their home cages. All procedures were performed under clean surgical condition.

### Tracer Injection for Dual Retrograde Tracing

**Figure 1:**
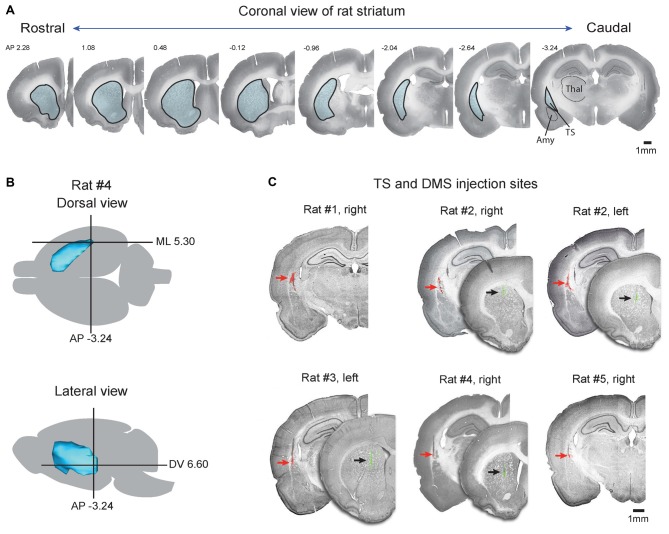
Injection sites of retrograde tracers in the tail of the striatum (TS) and dorsomedial striatum (DMS). **(A)** Coronal slices of Nissl-stained striatum in the rostral-caudal axis. Striatum outlines are indicated by black lines filled with blue. TS, tail of striatum; Amy, amygdala; Thal, thalamus. **(B)** Target coordinates of the injection site in the striatum. The striatum of rat #4 was reconstructed in the dorsal and lateral views, and coordination of the TS was marked by a cross based on the rat brain atlas (Paxinos and Watson, 1997). **(C)** Coronal view of the TS and DMS showing the red and green retrobead injection sites respectively. Fluorescent signals of red and green retrobeads were overlaid on Nissl-stained brain slices. Red and black arrows indicate the injection sites.

Anesthetized rats were placed in a stereotaxic frame, and the scalp was incised approximately 3 cm in length to expose the skull. We then drilled holes with 2-mm diameter in the skull to lower the injection needle (drill bit diameter: 1.35 mm). Red and green fluorescent retrobeads (Lumafluor) were used to compare TS-projecting neurons with DMS-projecting neurons in brain structures. Different fluorescent retrobeads were injected in the same rats to examine co-labeled neurons in the same brain slices. The injection sites and amounts are summarized in Table 1. 0.2 μl and 0.4 μl of red and green retrobeads were injected into the TS and DMS, respectively, except the first rat in Table 1. 0.5 μl of red retrobead was injected into the TS of rat #1, but we included the data of rat #1 because no significant differences were found in the distribution of labeled neurons compared to others. Among nine rats injected with the tracers, we chose the rats in which the retrograde tracers were selectively located in the target regions to reduce erroneous labeling (five rats in Figure 1C). We did not further analyze with the rest four rats. For retrobead injections, we used a silica tube (outer/inner tip diameter: 155/75 μm; Polymicro Technologies) attached to a 23-gauge stainless steel needle. After loading the retrobeads into a 10-μL Hamilton syringe, the injection needle was lowered slowly to the target region and held for 5 min to stabilize its position in the brain. The retrobeads were injected at a rate of 2 nl/s using a manual infusion pump (Stoelting). After 0.1 μl of retrobeads was injected, the needle was left for 1 min to minimize tracer diffusion along the needle track. After 0.2–0.5 μl was completely injected at one site, the needle was held for 10 min and drawn back slowly.

**Table 1:** Injection sites for cell counting.

	Injection sites for cell counting	Retrobead	Injection volume (μl)
Rat #1, TS	Right	Red	0.5
Rat #2, TS	Right	Red	0.2
DMS	Right	Green	0.4
Rat #2, TS	Left	Red	0.2
DMS	Left	Green	0.4
Rat #3, TS	Left	Red	0.2
DMS	Left	Green	0.4
Rat #4, TS	Right	Red	0.2
DMS	Right	Green	0.4
Rat #5, TS	Right	Red	0.2

### Histology

Seven to 10 days after injection, the rats were anesthetized with 5% isoflurane in oxygen for 3 min. For deep anesthesia, urethane (1.5 g in 5 ml saline) was intraperitoneally injected. To fix the brains, 250 ml of saline was perfused transcardially followed by the same volume of 4% paraformaldehyde. The brains were post-fixed overnight at 4°C and cryoprotected with 30% sucrose in phosphate-buffered saline (PBS; pH 7.4). The processed brains were frozen in OCT compound at −80°C and keep in the freezer overnight. The next day, the brains were sectioned with a cryostat (Leica CM1950) at 50-μm thickness, and every 200 μm (rat #2 and #5) and 400 μm (rat #1, #3 and #4) slices were used for cell counting. Images of brain slices were obtained with a fluorescent microscope (Leica DMi8). Adjacent slices were used for Nissl staining and immunohistochemistry to define brain structures and cell types, respectively.

### Immunohistochemistry

To examine whether the TS-projecting neurons were dopaminergic or serotonergic, the SN slices of rat #2, #3 and #4 were labeled with tyrosine hydroxylase (TH) antibody, and the dorsal raphe nucleus (DRN) slices of rat #2, #3 and #4 were labeled with serotonin (5-hydroxytryptamine) or TH antibodies. After 30 min permeabilization by 0.5% Triton X-100 in PBS, the slices were blocked with 3% normal goat serum and 2% bovine serum albumin in PBS (pH 7.4). After blocking, the SN slices were incubated with mouse anti-TH antibody (1:1000; Immunostar), and the adjacent slices (50-μm interval) containing the DRN were separately incubated with rabbit anti-serotonin antibody (1:500; Sigma) or mouse anti-TH antibody (1:1000; Immunostar) overnight at room temperature. On the second day, the slices were washed three times with PBS and incubated with secondary antibodies for 2 h at room temperature. Goat anti-mouse IgG antibody conjugated with Alexa 647 (1:350; Invitrogen) was used as secondary antibody for TH staining in the SN. The donkey anti-rabbit IgG antibody conjugated with Alexa 488 (1:350; Invitrogen) and goat anti-mouse IgG antibody conjugated with Alexa 488 (1:350; Invitrogen) were used for serotonin and TH staining in the adjacent slices containing the DRN, respectively. After three washes with PBS, the slices were air-dried overnight at room temperature and mounted with VECTASHIELD (Vector). Cell images of the SN and DRN slices were scanned with a super resolution confocal microscope (Leica DLS).

### Data Analysis

**Figure 2:**
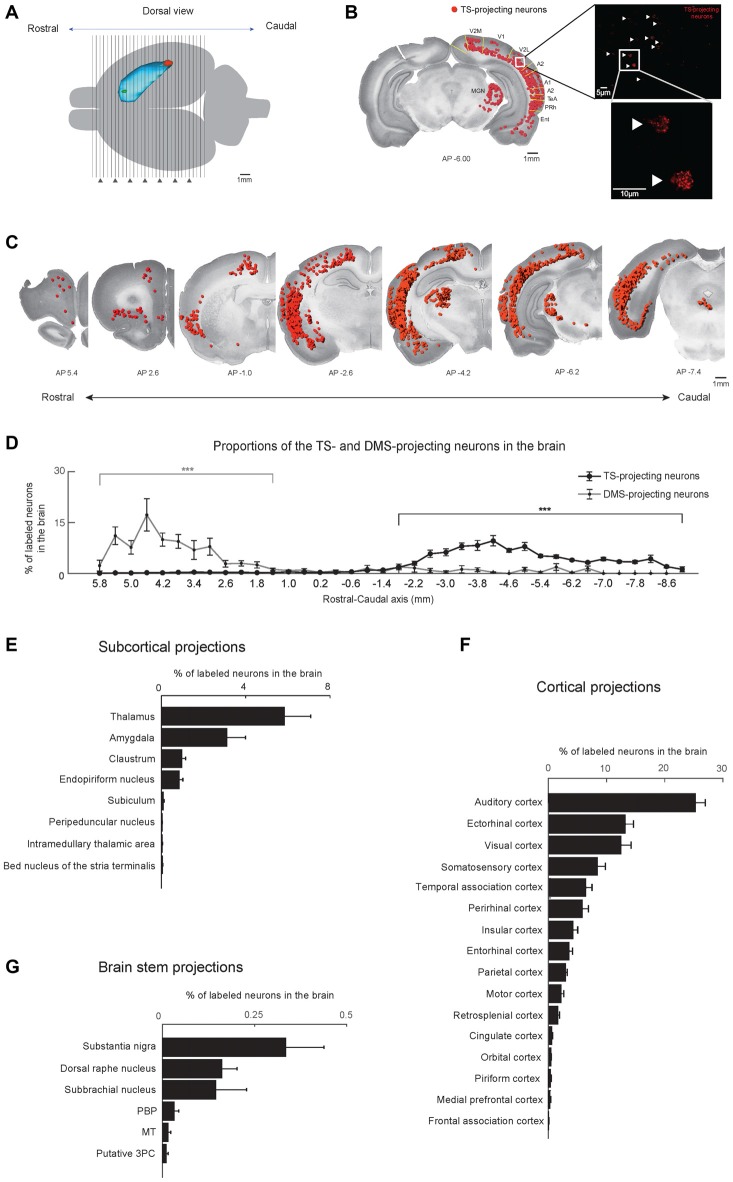
Cortical, subcortical and brain stem projections to the TS and DMS. **(A)** Dorsal view of the striatum showing red and green retrobead injection sites in the TS and DMS of rat #4. **(B)** Retrogradely labeled neurons from the TS injection site in a coronal brain section. The fluorescent signals were mostly found in the soma of neurons (right). These labeled neurons are marked as red dots and overlaid on a Nissl-stained brain section (left). V2M, medial part of the secondaryvisual cortex; V1, primary visual cortex; V2L, lateral part of the secondary visual cortex; A2, secondary auditory cortex; A1, primary auditory cortex; TeA, temporal association cortex; PRh, perirhinal cortex; Ent, entorhinal cortex. **(C)** TS-projecting neurons on coronal slices across the rostral-caudal axis. **(D)** Distributions of TS- and DMS-projecting neurons at 400-μm intervals in the rostral-caudal axis (TS-projecting neurons, *n* = 6 hemispheres; DMS-projecting neurons, *n* = 4 hemispheres). **(E–G)** Proportions of TS-projecting neurons in the cortical, subcortical, and brain stem regions (*n* = 6 hemispheres). TS-projecting neurons were mainly found in the midbrain, at ventral to the cerebral aqueduct, and dorsal to the medial longitudinal fasciculus, which may be the parvicellular part of the oculomotor nucleus (putative 3PC). PBP, parabrachial pigmented nucleus of the VTA; MT, medial terminal nucleus of the accessory optic tract. ***p < 0.001.

To reconstruct the striatum of rats injected with retrograde tracers (Figures 1B, 2A), the outlines of the cortex, striatum and fluorescent signals of injected retrograde tracers were drawn through 400-μm interval brain slices, and the images were reconstructed with IMOD, a 3D rendering program (Boulder Laboratory, University of Colorado, Boulder, CO, USA). To count the labeled cells, we scanned at 200-μm intervals from three hemispheres (rat #2 right, left and rat #5) and at 400-μm intervals from three hemispheres (rat #1, #3 and #4). Labeled cells and brain structure outlines in the scanned images were analyzed and marked in Adobe Illustrator CS6 based on the outlines of Nissl-stained slices and the rat brain atlas (Paxinos and Watson, 1997).

**Figure 3:**
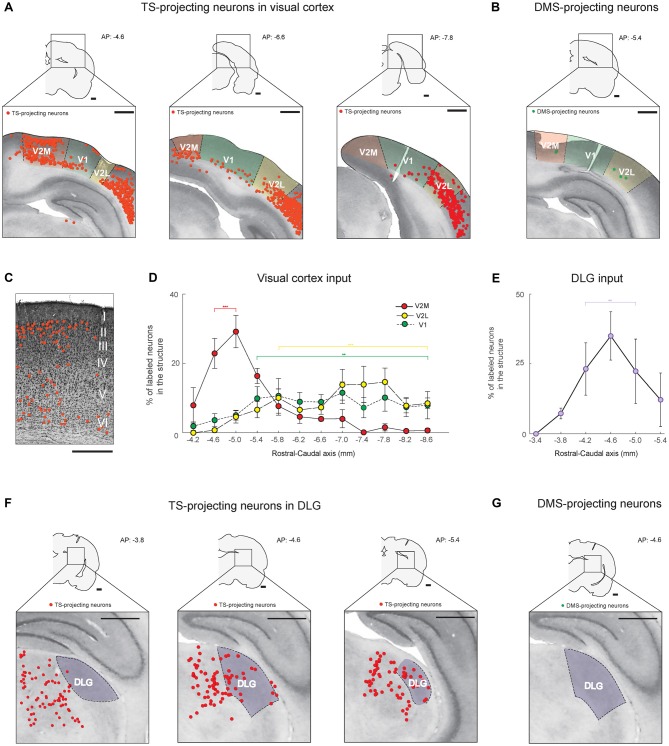
The TS receives cortical and thalamic visual inputs: visual cortex and dorsolateral geniculate nucleus (DLG) projections. **(A)** TS-projecting neurons in the rostral, middle and caudal parts of the visual cortex. V1, primary visual cortex; V2L, lateral part of the secondary visual cortex; V2M, medial part of the secondary visual cortex. **(B)** DMS-projecting neurons were found in the visual cortex. Scale bars: 1 mm.** (C)** An example of Nissl-stained slice showing the location of TS-projecting neurons in the V2M layers (Rat #2, left side of the brain). Scale bar: 500 μm. **(D)** Rostral-caudal distribution of TS-projecting neurons at 400-μm intervals in the subregions of the visual cortex (*n* = 6 hemispheres). **(E)** Rostral-caudal distribution of TS-projecting neurons at 400-μm intervals in the DLG (*n* = 6 hemispheres). **(F)** TS-projecting neurons in the rostral, middle and caudal parts of the DLG. **(G)** No DMS-projecting neurons were found in the DLG. Scale bars: 1 mm. **p < 0.01,***p < 0.001.

**Figure 4:**
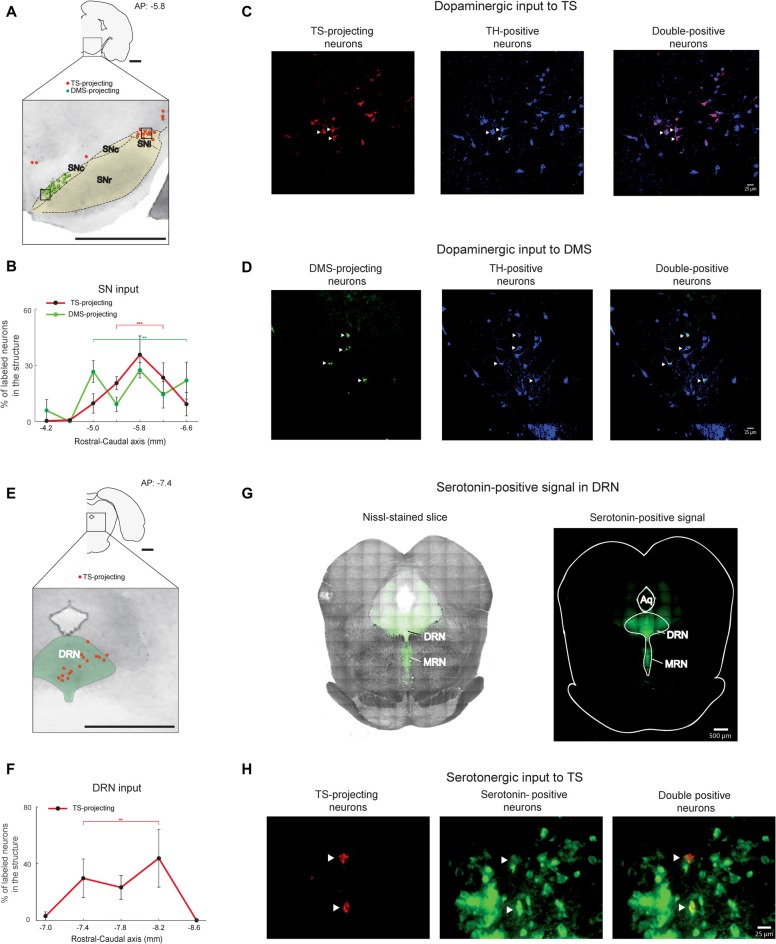
SN and DRN inputs to the TS and DMS. **(A)** Retrogradely labeled neurons in and around the SN (black dotted line). TS-projecting neurons (red dots) were located in the lateral region of the SN, mostly in the substantia nigra pars lateralis (SNl). DMS-projecting neurons (green dots) were mostly found in the medial region of the SN. SNr, substantia nigra pars reticulata; SNc, Substantia nigra pars compacta. Scale bar: 1 mm. **(B)** Rostral-caudal distributions of TS- and DMS-projecting neurons at 400-μm intervals in the SN (*n* = 6 hemispheres and *n* = 4 hemispheres, respectively). **p < 0.01, ***p < 0.001. **(C,D)** TS- and DMS-projecting neurons in the SN were dopaminergic. Signals of retrogradely labeled neurons (left) colocalized with tyrosine hydroxylase (TH)-positive signals (middle). Merged images (right) show double-labeled neurons (arrow heads in example). Blue is pseudocolor for TH-positive cells. Scale bars: 25 μm. **(E)** TS- projecting neurons in the DRN. DMS-projecting neurons were not found in the slice. The green-labeled area indicates the DRN. DRN, dorsal raphe nucleus. Scale bar: 1 mm. **(F)** Rostral-caudal distribution of TS-projecting neurons at 400-μm intervals in the DRN (*n* = 6 hemispheres). **p < 0.01. **(G)** Serotonin-positive signals (right) were overlaid on the Nissl-stained brain stem (left). MRN, median raphe nucleus. Scale bars: 500 μm. **(H)** TS-projecting neurons in the DRN were serotonergic. Retrogradely labeled signals (left) were colocalized with serotonin-positive signals (middle). Merged images (right) show double-labeled neurons (arrow heads). Scale bar: 25 μm. **p < 0.01, ***p < 0.001.

**Figure 5:**
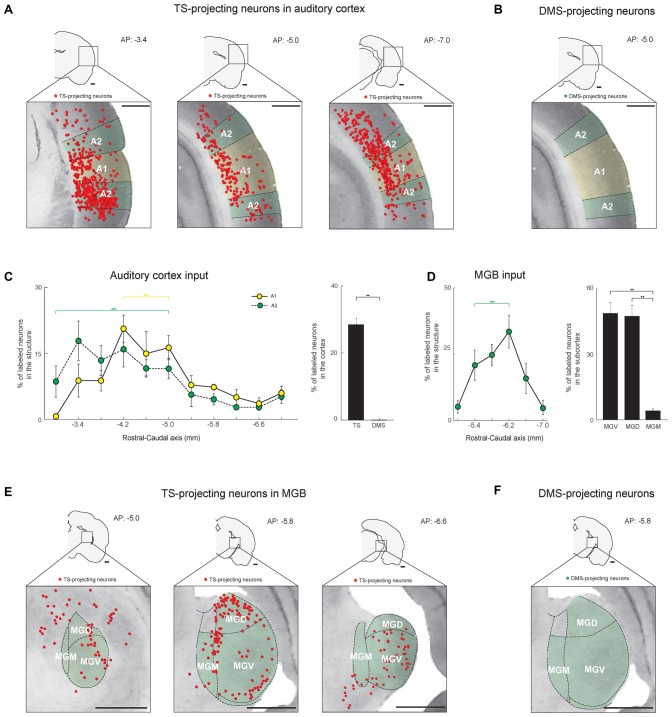
The TS receives cortical and thalamic auditory inputs: auditory cortex and medial geniculate body (MGB) projections. **(A)** TS-projecting neurons in the rostral, middle, and caudal parts of the auditory cortex. A1, primary auditory cortex; A2, secondary auditory cortex. **(B)** DMS-projecting neurons were not found in the auditory cortex. **(C)** Rostral-caudal distribution of TS-projecting neurons at 400-μm intervals in the subregions of the auditory cortex (left) and proportions of projecting neurons in the cortex (right; TS-projecting neurons, *n* = 6 hemispheres; DMS-projecting neurons, *n* = 4 hemispheres). **(D)** Rostral-caudal distribution of TS-projecting neurons at 400-μm intervals in the MGB (left) and their proportions in the MGB subregions (right; TS-projecting neurons, *n* = 6 hemispheres). MGB, medial geniculate body; MGV, ventral part of the MGB; MGD, dorsal part of the MGB; MGM, medial part of the MGB. **(E)** TS-projecting neurons in the rostral, middle, and caudal parts of the MGB. **(F)** No DMS-projecting neurons were found in the MGB. Scale bars: 1 mm. **p < 0.01,***p < 0.001.

**Figure 6:**
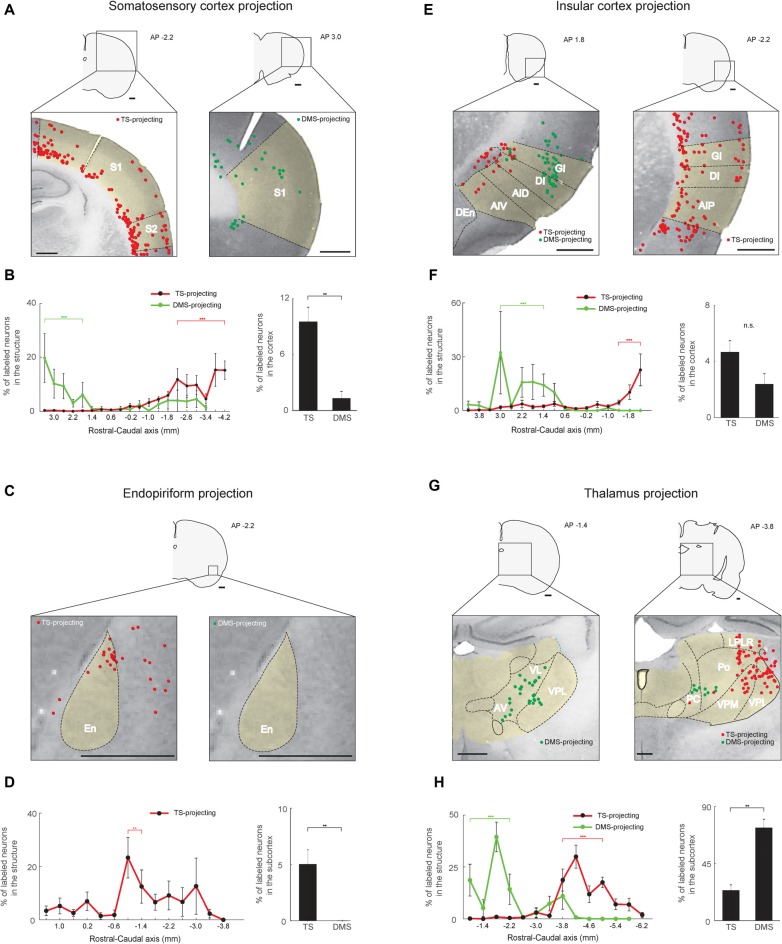
Somatosensory, gustatory and olfactory inputs to the TS and DMS from cortical and thalamic regions. **(A)** TS- and DMS-projecting neurons (red and green dots, respectively) in the somatosensory cortex. S1, primary somatosensory cortex; S2, secondary somatosensory cortex. **(B)** Rostral-caudal distributions of TS- (red line) and DMS-projecting (green line) neurons at 400-μm intervals in the somatosensory cortex (left) and proportions of projecting neurons in the cortex (right; TS-projecting neurons, *n* = 6 hemispheres; DMS-projecting neurons, *n* = 4 hemispheres). **(C,D)** The same format as in **(A,B)**, showing projections from the endopiriform cortex (En). **(E,F)** The same format as in **(A,B)**, showing inputs from the insular cortex. GI, granular insular cortex; DI, dysgranular insular cortex; AID, dorsal part of the agranular insular cortex; AIV, ventral part of the agranular insular cortex; AIP, posterior part of the agranular insular cortex. **(G,H)** The same format as in **(A,B)**, showing projections from the thalamus. VL, ventrolateral thalamic nucleus; AV, anteroventral thalamic nucleus; LPLR, lateral part of the lateral posterior thalamic nucleus; PC, paracentral thalamic nucleus; Po, posterior thalamic nuclear group; VPM, ventral posteromedial thalamic nucleus (medial part of the VPN); VPL, ventral posterolateral thalamic nucleus (lateral part of the VPN). Scale bars: 1 mm. **p < 0.01, ***p < 0.001.

**Figure 7:**
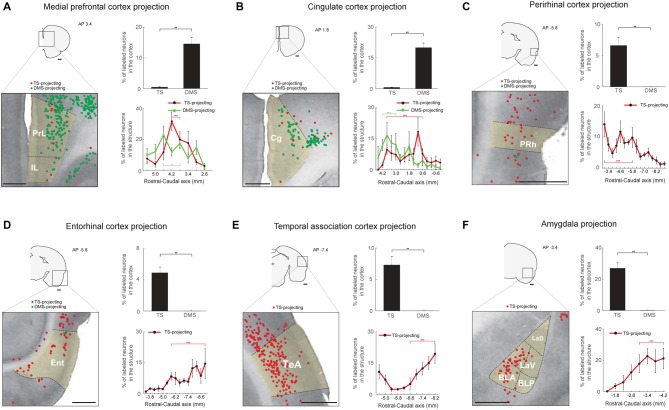
Limbic inputs to the TS and DMS. **(A)** TS- and DMS-projecting neurons (red and green dots, respectively) in the medial prefrontal cortex (mPFC; left) and proportions in the cortex (top-right). Rostral-caudal distributions of TS- (red line) and DMS-projecting (green line) neurons at 400-μm intervals in the mPFC cortex (bottom-right; TS-projecting neurons, *n* = 6 hemispheres; DMS-projecting neurons, *n* = 4 hemispheres). PrL, prelimbic cortex; IL, infralimbic cortex. **(B)** The same format as in **(A)** showing cingulate cortex (Cg) inputs. **(C)** The same format as in **(A)**, showing perirhinal cortex (PRh) inputs. **(D)** The same format as in **(A)**, showing entorhinal cortex (Ent) inputs. **(E)** The same format as in **(A)**, showing temporal association cortex (TeA) inputs. **(F)** The same format as in **(A)**, showing amygdala input proportions in the subcortex. BLA, anterior part of the basolateral amygdaloid nucleus; BLP, posterior part of the basolateral amygdaloid nucleus; LaV, ventral part of the lateral amygdaloid nucleus; LaD, dorsal part of the lateral amygdaloid nucleus. Scale bars: 1 mm. **p < 0.01, ***p < 0.001.

**Figure 8:**
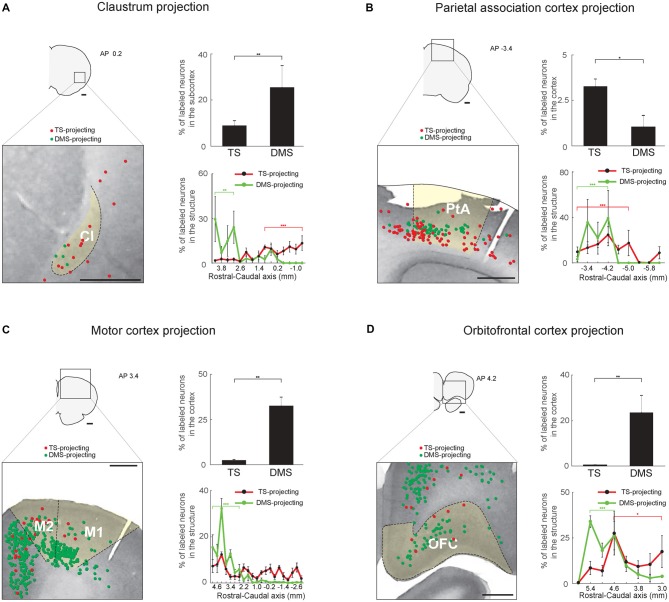
Inputs from the sensory associative structures, motor cortex and orbitofrontal cortex (OFC) to the TS and DMS. **(A)** TS- and DMS-projecting neurons (red and green dots, respectively) in the claustrum (Cl; left) and their proportions in the subcortex (top-right). Rostral-caudal distributions of TS- (red line) and DMS-projecting (green line) neurons at 400-μm intervals in the Cl (bottom-right; TS-projecting neurons, *n* = 6 hemispheres; DMS-projecting neurons, *n* = 4 hemispheres). **(B)** The same format as in **(A)**, showing parietal association cortex (PtA) inputs. **(C)** The same format as in **(A)**, showing motor cortex inputs. M1, primary motor cortex; M2, secondary motor cortex. **(D)** The same format as in **(A)**, showing OFC inputs. Scale bars: 1 mm. *p < 0.05, **p < 0.01, ***p < 0.001.

To identify the brain structures that innervate the TS and DMS, we confirmed that the TS- and DMS-projecting neurons in each brain structure were consistently found in all six and four hemispheres, respectively. These labeled neurons in the confirmed brain structures were used for further analysis described below. To examine the rostral-caudal distributions of labeled neurons in the whole brain and brain structures, the number of neurons in each coronal slice was divided by the number of labeled cells in the whole brain (Figure 2D and Supplementary Figure S1A, line graphs, and Figures 2E–G, bar graphs) or in a certain structure (Figures 3–8, line graphs, and Supplementary Figures S2, S4–S6).

Each percentage of labeled neurons in the rostral-caudal axis was calculated with counted numbers of TS-projecting neurons in six hemispheres and DMS-projecting neurons in four hemispheres. The percentages of TS- and DMS-projecting neurons were plotted at every 400-μm intervals from six and four hemispheres, respectively, in main figures. To further confirm the distributions of projection neurons with a narrower interval, we plotted the percentages of TS- and DMS-projecting neurons at every 200-μm intervals from 3 (rat #2, right, left and rat #5) and two hemispheres (rat #2, right and left), respectively, in Supplementary Figures. To analyze the labeled cell distribution in the rostral-caudal axis of each structure, we compared the percentage data in chosen series of the brain slices to the percentages in rest of the brain slices using Wilcoxon rank-sum test with MATLAB software (Mathworks Inc., Natick, MA, USA).

To compare the proportions of TS- and DMS-projecting neurons in a structure, the number of retrogradely labeled neurons was divided by the total number of labeled neurons in the cortex, subcortex and brain stem (Figures 5–8, bar graphs). Statistical significance was examined with Wilcoxon rank-sum test. The data were plotted with MATLAB software (Mathworks Inc., Natick, MA, USA).

## Results

### The Tail of the Striatum in Rat Brain and Retrograde Tracer Injection

The TS is located in the caudal portion of the rat striatum. It is relatively small but continuous with the rest of the striatum and can be clearly identified in Nissl-stained sections, as shown in Figure 1A. In coronal sections, the TS is surrounded by the external and internal capsules and is distinct from other structures, such as the amygdala, thalamus, and cortex (Figure 1A, caudal planes). We injected red retrobeads into the TS using brain atlas coordinates (Anterior-Posterior (AP) = −3.24, ML = 5.30, Dorsal-Ventral (DV) = 6.60; Figure 1B; Table 1, Paxinos and Watson, 1997). In each rat, these injections were in the caudal-most part of the striatum above the amygdala (Figures 1A,C). To compare the TS and DMS inputs, we injected green retrobeads into the DMS of the same rats using brain atlas coordinates (AP = 1.08, ML = 2.4, DV = 5.0; Figure 2A; Table 1).

To examine the exact location of each injection site, fluorescent signals of injected retrobeads were overlaid on Nissl-stained coronal slices (Figure 1C). Among the nine rats injected with the tracers, we chose and analyzed the three rats in which injected red and green retrobeads were successfully located in the TS and DMS, respectively (Rat #2, #3, and #4 in Figure 1C). In addition, we also analyzed the two rats in which injected red retrobeads were successfully located only in the TS (Rat #1 and #5 in Figure 1C). Fluorescent signals of red retrobeads were mainly found in the dorsal region of the TS. The green retrobead injection sites were also selectively localized in the DMS of three out of five rats (Figure 1C). Seven to 10 days after the injection, we sacrificed the rats to examine the labeled neurons in the cortex, subcortex and brain stem.

### Topographic Projections to the TS and DMS in the Rostral-Caudal Axis of the Brain

Coronal brain sections (50 μm thickness) of retrobead-injected rats were examined at 400-μm and 200-μm intervals (Figure 2A). We observed fluorescent retrobeads in the soma of neurons (Figure 2B, right). The labeled neurons were localized to specific regions of the cortex, subcortex and brain stem (Figure 2B, left and Figure 2C).

We found more TS-projecting neurons in the brain structures located in caudal regions of the cortex, subcortex and brain stem (*P* < 0.001, Wilcoxon rank-sum test; Figures 2C,D and Supplementary Figure S1). In contrast, more DMS-projecting neurons were found in the brain structures located in rostral regions of the cortex, subcortex and brain stem (*P* < 0.001, Wilcoxon rank-sum test), agreeing with previous research (Hintiryan et al., 2016). This neuronal distribution showed topographic projections to the TS and DMS from the rostral-caudal axis.

The TS received inputs from the cortex, subcortex and the brain stem (Figures 2E–G). The cortical regions including sensory-processing areas innervated the TS (Figure 2F). In the subcortex, the TS mainly received inputs from the thalamus, amygdala, claustrum (Cl) and the endopiriform nucleus (Figure 2E). Three regions in the brain stem mainly projected to the TS: the substantia nigra, dorsal raphe nucleus, and subbrachial nucleus (Figure 2G). Among the brain inputs, we first examined the cortical and subcortical inputs involved in sensory processing and neuromodulation.

### Cortical and Thalamic Visual Inputs to the TS

Neurons in the visual cortex projected to the TS, as previously reported in anterograde and retrograde tracing studies (Khibnik et al., 2014; Hintiryan et al., 2016), but the TS-projecting neurons were mostly localized in the V2 area (Figure 3A). The retrogradely labeled neurons from the TS injection site were found in the layers 5/6 of V1 and V2 and specifically in the layers 2/3 of rostral V2M (Figure 3A, left and Figure 3C). In V2M, they were more numerous in the rostral than in the caudal part (*P* < 0.001, Wilcoxon rank-sum test). In V1 and V2L, we found more TS-projecting neurons in the central-caudal part than the rostral part (*P* < 0.01 and *P* < 0.001, Wilcoxon rank-sum test, respectively; Figure 3D and Supplementary Figure S2A). We then compared the differences in visual inputs to the TS and DMS using the rats injected with both red and green retrobeads (four hemispheres in three rats; an example in Figure 2A). We found few or no labeled neurons from the DMS injection site in the visual cortex (Figure 3B), indicating that the TS receives strong input from the visual cortex while the DMS receives weak visual input.

We found inputs from a thalamic region known to be important for visual processing. Retrogradely labeled neurons from the TS injection site were located in the dorsolateral geniculate nucleus (DLG; Figure 3F). These labeled neurons were distributed differently within the nucleus. We found more TS-projecting neurons in the central and caudal part of the DLG than in the rostral part (*P* < 0.01, Wilcoxon rank-sum test; Figures 3E,F and Supplementary Figure S2B). No neurons in the DLG were labeled from the DMS injection site (Figure 3G). These data showed that the TS receives numerous visual input from V1, V2 and the DLG.

### Dopaminergic and Serotonergic Inputs to the TS

The visual inputs to the TS suggest that the caudal region of the striatum is involved in visual information processing. If the same region of the TS received value information from other brain regions, it would suggest that TS is important for associating visual and value information, such as for visual habit. Therefore, we investigated whether value-coding regions innervate the TS.

We found that the TS received inputs from the SN and DRN, two brain stem regions known to encode reward information (Tobler et al., 2005; Nakamura et al., 2008; Kim et al., 2015; Li et al., 2016). In primate studies, dopamine neurons in the SN were one of the main inputs to the CDt, which might be homologous to the rat TS (Kim et al., 2014, 2015). Dopamine neurons in the caudal-dorsal-lateral region of the SN pars compacta (cdlSNc) mainly innervated the CDt, whereas dopamine neurons in the rostral-ventral-medial region of the SNc (rvmSNc) innervated the CDh (Kim et al., 2014). In the rat SN, we found the most labeled neurons within the ipsilateral dorsal-lateral SN, mostly in the SN pars lateralis (SNl; Figure 4A). These TS-projecting neurons were clearly segregated from DMS-projecting neurons. TS-projecting neurons were found in the dorsal-lateral region, whereas DMS-projecting neurons were in the ventral-medial region of the SN (Figure 4A). Both TS- and DMS-projecting neurons in the SN were differentially distributed across the rostral-caudal axis, more from the central-caudal part (*P* < 0.001 and *P* < 0.01, respectively, Wilcoxon rank-sum test; Figure 4B and Supplementary Figure 2C). To confirm that the TS- and DMS-projecting neurons in the SN were dopaminergic, we identified TH-positive cells using immunohistochemistry. The TS- and DMS-projecting neurons in the SN were double-labeled with anti-TH antibody (Figures 4C,D), indicating that these TS- and DMS-projecting neurons in the SN were dopaminergic. We found that 85.3 ± 5.4% of TS-projecting neurons in the SN was double-labeled with anti-TH antibody (*n* = 3 rats; #2, #3 and #4).

TS-projecting neurons were found in the midline and ipsilateral regions of the DRN (Figure 4E). These TS-projecting neurons were more numerous in the central region (*P* < 0.01, Wilcoxon rank-sum test; Figure 4F and Supplementary Figure S2D). Neurons in the DRN were not labeled from the DMS injection site. To identify whether or not TS-projecting neurons were serotonergic, we labeled the DRN slices containing the TS-projecting neurons with an anti-serotonin antibody that targeted the cell body and ascending fibers of serotonergic neurons in dorsal and median raphe nucleus (DRN and MRN; Figure 4G). The retrogradely labeled signals from the TS injection site and the signals from anti-serotonin antibody were colocalized within the cell bodies of DRN neurons (Figure 4H), indicating that the TS-projecting neurons in the DRN were serotonergic. 74.6 ± 13.0% of TS-projecting neurons in the DRN was double-labeled with anti-serotonin antibody (*n* = 3 rats; #2, #3 and #4).

The DRN is also known to contain TH-positive neurons (Trulson et al., 1985; Stratford and Wirtshafter, 1990). To identify whether these TS-projecting neurons in the DRN are TH-positive, we labeled DRN slices containing the TS-projecting neurons with anti-TH antibody. Retrogradely labeled neurons from the TS injection site were not labeled with the TH-antibody in the DRN (Supplementary Figure S3), suggesting that these TS-projecting neurons in the DRN are not dopaminergic.

### Cortical and Thalamic Auditory Inputs to the TS

Similar to the visual inputs, we found auditory inputs from the auditory cortex and a thalamic area, the medial geniculate body (MGB), to the TS (Figures 5A,E). In the auditory cortex, TS-projecting neurons were found in the A1 and A2 regions and were more numerous in the rostral-central parts (*P* < 0.001 and *P* < 0.001, respectively, Wilcoxon rank-sum test; Figure 5C, left and supplementary Figure S2E). No neurons in the auditory cortex were labeled from DMS injection site (Figures 5B,C, right).

In the thalamus, TS-projecting neurons were found in the MGB and were more numerous in the rostral-central region (*P* < 0.001, Wilcoxon rank-sum test; Figures 5E,D-left and Supplementary Figure S2F). Unlike the rodent LGN, in which the subregions are not clearly divided (Van Hooser and Nelson, 2006), the rodent MGB is largely divided into three subregions: dorsal (MGD), ventral (MGV), and medial (MGM; Clerici and Coleman, 1990; Winer et al., 1999). TS-projecting neurons were found in all three subregions but were more prevalent in the MGV and MGD than MGM (*P* < 0.01 and *P* < 0.01, respectively, Wilcoxon rank-sum test; Figure 5D, right). The projecting neurons were located around the edges of the subregions, which might include the marginal zone of the MGB (Figure 5E, middle). No MGB neurons were labeled from the DMS injection site (Figure 5F). These data together with the visual inputs to the TS show innervations from auditory cortical and thalamic structures to the TS.

### Somatosensory-, Olfactory- and Gustatory-Related Cortical and Thalamic Inputs to the TS

Our findings of visual and auditory inputs to the TS led us to examine inputs from other sensory areas to the TS. The TS received inputs from sensory cortices involved in somatosensory, olfactory, and gustatory information processing. Within the somatosensory cortex, these TS-projecting neurons were mostly found in deep layers (5/6), although some were found in layer 2 (Figure 6A, left). In the rostral-caudal axis, we found most TS-projecting neurons in the caudal part of the somatosensory cortex (*P* < 0.001, Wilcoxon rank-sum test) and most DMS-projecting neurons in the rostral part (*P* < 0.001, Wilcoxon rank-sum test; Figure 6B, left and Supplementary Figure S4A). We found fewer DMS-projecting neurons than TS-projecting neurons in the somatosensory cortex (*P* < 0.01, Wilcoxon rank-sum test; Figure 6B, right).

TS-projecting neurons were found in the endopiriform area, an area involved in processing olfactory and gustatory information (Fu et al., 2004; Oliveira-Maia et al., 2011; Courtiol and Wilson, 2015). We found no endopiriform neurons labeled from the DMS injection site (*P* < 0.01, Wilcoxon rank-sum test; Figures 6C,D, right). The TS-projecting neurons were more numerous in the central part of the endopiriform area (*P* < 0.01, Wilcoxon rank-sum test; Figure 6D, left and Supplementary Figure S4C).

TS-projecting neurons were found in the insular cortex (Figure 6E), which is involved in various functions including gustatory, auditory, somatosensory, and pain processes (de Araujo et al., 2012; Moraga-Amaro and Stehberg, 2012). The insular cortex contained similar numbers of neurons labeled from the TS and DMS injection sites (*P* = 0.114, Wilcoxon rank-sum test; Figure 6F, right), but the layer and rostral-caudal distributions of these labeled neurons were different. TS-projecting neurons were found in deeper layers (5/6) than the DMS-projecting neurons (layers 2/3; Figure 6E, left). TS-projecting neurons were more numerous in the caudal part of the insular cortex (*P* < 0.001, Wilcoxon rank-sum test), while the DMS-projecting neurons tended to be located in the rostral-central part (*P* < 0.001, Wilcoxon rank-sum test; Figure 6F, left and Supplementary Figure S4B).

In the rest of the thalamus except the DLG and MGB, we found TS-projecting neurons. We found fewer TS-projecting neurons than DMS-projecting neurons (*P* < 0.01, Wilcoxon rank-sum test; Figure 6H, right). These TS-projecting neurons were mostly located in the caudal regions of the thalamus (*P* < 0.001, Wilcoxon rank-sum test), while the DMS-projecting neurons were mostly located in the rostral region (*P* < 0.001, Wilcoxon rank-sum test; Figures 6G,H-left, and Supplementary Figure S4D). TS-projecting neurons were mainly found in the ventral posterior thalamic nucleus (VPN), which has functions in somatosensory and gustatory processes (Figure 6G, right; Jones et al., 1986; Landisman and Connors, 2007; Oliveira-Maia et al., 2011). These data indicate that the cortical and thalamic regions involved in somatosensory, olfactory and gustatory processes innervated the TS.

### Limbic Structures Innervate the TS

We found cortical and subcortical limbic structures that projected to the TS. First, structures in the limbic cortex innervated the TS and DMS. We found more DMS-projecting neurons than TS-projecting neurons in the medial prefrontal (mPFC) and cingulate cortex (*P* < 0.01 and *P* < 0.01, respectively, Wilcoxon rank-sum test; Figures 7A,B top-right). TS- and DMS-projecting neurons were more numerous in the central region of mPFC (*P* < 0.001 and *P* < 0.05, respectively, Wilcoxon rank-sum test; Figure 7A, bottom-right and Supplementary Figure S5A). In the cingulate cortex, neurons in the rostral part more innervated the DMS (*P* < 0.001, Wilcoxon rank-sum test), and TS-projecting neurons were more located in the central part (*P* < 0.001, Wilcoxon rank-sum test; Figure 7B, bottom-right and Supplementary Figure S5B). The perirhinal cortex (PRh), entorhinal cortex, and temporal association cortex (TeA) selectively projected to the TS (*P* < 0.01, *P* < 0.01, and *P* < 0.01, respectively, Wilcoxon rank-sum test; Figures 7C–E, top-right panels). TS-projecting neurons were mainly localized to the caudal region of each structure (Entorhinal cortex: *P* < 0.001; TeA: *P* < 0.001, Wilcoxon rank-sum test; Figures 7D,E, bottom-right, and Supplementary Figures S5D,E), except in the PRh where TS-projecting neurons were more localized to the rostral part (*P* < 0.001, Wilcoxon rank-sum test; Figure 7C, bottom-right and Supplementary Figure S5C).

Second, the amygdala in the subcortical limbic system strongly innervated the TS (Figure 7F). Retrogradely labeled neurons from the TS injection site were found in the lateral amygdala, but no labeled neurons were found from the DMS injection site (*P* < 0.01, Wilcoxon rank-sum test; Figure 7F, top-right). These TS-projecting neurons were found in the caudal part of the amygdala, indicating that neurons in this caudal part more strongly innervated the TS (*P* < 0.001, Wilcoxon rank-sum test; Figure 7F, bottom-right and Supplementary Figure S5F).

### Multisensory Regions Innervate the TS: Inputs From the Claustrum and Parietal Association Cortex

Two regions, the Cl and parietal association cortex (PtA), might process various sensory inputs (Torrealba and Valdés, 2008; Goll et al., 2015). Both TS- and DMS-projecting neurons were found in these structures, and DMS received more inputs from the Cl than TS whereas TS received more inputs from the PtA than DMS (*P* < 0.01 and *P* < 0.05, respectively, Wilcoxon rank-sum test; Figures 8A,B, top-right). In the Cl, the TS- and DMS-projecting neurons were differently distributed in the rostral-caudal axis; TS- and DMS-projecting neurons were more localized to the caudal and rostral regions, respectively (*P* < 0.001 and *P* < 0.01, respectively, Wilcoxon rank-sum test; Figure 8A, bottom-right and Supplementary Figure S6A). In the PtA, TS- and DMS-projecting neurons were both found in the deep layers (5/6) and their distribution showed layer difference (Figure 8B, left). Both TS- and DMS-projecting neurons showed a weak but significant distribution difference. TS-projecting neurons were localized to the rostral-central and DMS-projecting neurons to the rostral region of the PtA (*P* < 0.001 and *P* < 0.001, respectively, Wilcoxon rank-sum test; Figure 8B, bottom-right and Supplementary Figure S6B).

### Sparse Inputs From the Motor Cortex to the TS

The DMS is known to receive inputs from the motor cortex (Hintiryan et al., 2016). However, relatively few TS-projecting neurons were found in the motor cortex in comparison to DMS-projecting neurons (*P* < 0.01, Wilcoxon rank-sum test; Figure 8C, top-right). The TS-projecting neurons were mostly intermingled with DMS-projecting neurons, but no neurons projecting to both TS and DMS were found (Figure 8C, left). We found more DMS-projecting neurons in the rostral part of the motor cortex than the caudal part (*P* < 0.001, Wilcoxon rank-sum test; Figure 8C, bottom-right and Supplementary Figure S6C).

### Weak Input From the Orbitofrontal Cortex to the TS

The orbitofrontal cortex (OFC) is an important structure in encoding reward value in primates and rodents (Gottfried et al., 2003; Sul et al., 2010; Rudebeck et al., 2013). Fewer TS-projecting than DMS-projecting neurons were found in the OFC (*P* < 0.01, Wilcoxon rank-sum test; Figure 8D, top-right). The TS-projecting neurons were mostly localized in the central-caudal region of the OFC (*P* < 0.05, Wilcoxon rank-sum test; Figure 8D, bottom-right and Supplementary Figure S6D). In contrast, the DMS received strong inputs from the OFC, and we found DMS-projecting neurons mostly in the rostral part of the OFC (*P* < 0.001, Wilcoxon rank-sum test; Figure 8D, bottom-right and Supplementary Figure S6D; Hintiryan et al., 2016).

## Discussion

**Figure 9:**
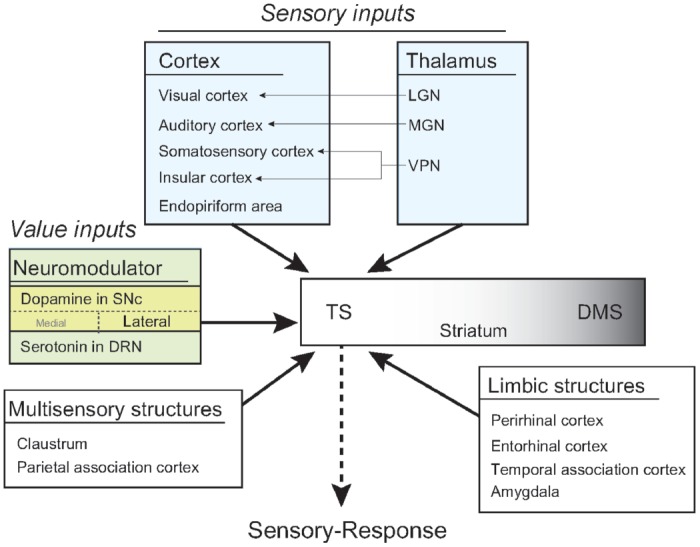
A model of habit learning in the TS by sensory and value inputs. The rat TS directly receives sensory and value inputs from the cortex, thalamus and brain stem. This anatomical convergence of sensory and value inputs in the TS allows for learning of a sensory-response association for habitual behavior. Furthermore, the thalamic sensory inputs might produce a stimulus-induced quick response after long-term learning.

Our anatomy study revealed that the rat TS received inputs from sensory, neuromodulatory, limbic, and associative systems in cortical and subcortical structures. Visual and dopaminergic regions innervated the rat TS (Figure 9), indicating that the TS is anatomically sufficient to encode the visual stimuli-behavioral response association for visual habit previously reported in primates (Kim and Hikosaka, 2013; Yamamoto et al., 2013). Furthermore, auditory, somatosensory, gustatory and olfactory regions in the cortex and the thalamus projected to the TS (Figure 9). This indicates that the TS is also anatomically sufficient for the association of general sensory stimuli and motor responses in habitual behavior.

### Topographical Projections to the TS

We showed clear topography of striatal inputs. More TS-projecting neurons were found in the brain structures located in caudal regions of the cortex, subcortex and brain stem, whereas more DMS-projecting neurons were found in the brain structures located in the rostral regions (Figure 2D and Supplementary Figure S1). Interestingly the different spatial distribution of the projection neurons was also found in the rostral-caudal axis of each brain structure. Half of the analyzed structures (10 out of 20 structures) had more TS-projecting neurons in their caudal parts. The other half of the analyzed structures had more TS-projecting neurons in their rostral or rostral-central parts (3 and 6 structures, respectively). This result indicates that subregions in one brain structure receive different inputs, suggesting their different functions along the rostral-caudal axis.

### Sensory Cortex and Sensory Thalamus Inputs to the TS

In the cortex, the secondary sensory regions more strongly innervated the TS than did the primary sensory regions, suggesting that processed information is sent to the TS. In addition, thalamic structures (LGN, MGB, and VPN) that innervate the primary sensory cortices directly projected to the TS (Alitto and Usrey, 2003). These thalamic structures are involved in low-level sensory processes. For example, a group of neurons in the dorsal MGB has differential responses to different sound frequencies, showing a tonotopic map (Alitto and Usrey, 2003; Shiramatsu et al., 2016). These anatomical connections suggest that the TS receives both processed and less-processed sensory inputs from the sensory cortices and the thalamic structures, respectively (Xiong et al., 2015). The thalamic inputs to the TS might allow for quick and automatic responses after long-term learning. A functional study of these sensory inputs from the cortex and thalamus will show the mechanisms of automatic behavior in future.

### Distinct Group of Dopamine Neurons Innervate the TS

Dopaminergic input is thought to be involved in synaptic plasticity of the basal ganglia system (Wickens, 2009). Dopamine neurons are quite heterogeneous in their functions and anatomical connections (Kim et al., 2014; Lerner et al., 2015; Menegas et al., 2015, 2017). A primate study found that distinct groups of dopamine neurons in the rostral-medial and caudal-lateral regions of the SNc innervated the head and tail of CD, respectively, and encoded flexible and stable values of visual objects (Kim et al., 2015). Here, we found the difference in spatial distribution of the TS- and DMS-projecting neurons in the ML axis, but no significant difference in the rostral-caudal axis (Figures 4A,B). TS-projecting dopamine neurons were mostly localized to the caudal-lateral part of SN, whereas DMS-projecting neurons were mostly localized to the caudal-medial part of the SN. This anatomical result showed that distinct groups of dopamine neurons might associate reward information with sensory information from cortical and thalamic structures (Figure 9). The different anatomical locations of TS- and DMS-projecting dopamine neurons may imply their functional differences. Indeed, a recent mouse study showed that TS-projecting dopamine neurons encode novelty signal for rewarding, aversive and neutral stimuli, which is different from the typical function of reward prediction error-coding dopamine neurons (Menegas et al., 2017). In addition, it is known that the mouse TS has a low expression level of the dopamine D2 receptor compared to other striatal regions (Gangarossa et al., 2013), suggesting that multi-level differences in dopaminergic input, function and expression level of dopamine receptor may generate a unique role of the TS.

### Serotonergic Projection to the TS

In addition to the SN dopaminergic input, serotonergic input to the TS was found in the DRN (Figures 4E–H). Different from the previous studies using autoradiographic anterograde tracers (Moore et al., 1978; Soghomonian et al., 1987), we did not find the DRN input to the DMS injection site. This might be due to the difference in projection strength to the TS and DMS or the region of DMS injection site. However, our finding is consistent with the recent monkey data, showing that the primate DRN has projections to the CDt but no identified projections to the CDh injection site (Griggs et al., 2017). Previous studies showed that tonic neuronal response in the DRN was modulated by the expected and received reward values (Nakamura et al., 2008; Li et al., 2016). This reward signal might be sent to the caudal part of the striatum to modulate synaptic strength.

### Weak Projection From the OFC to the TS

We also found a few neurons projecting to the TS in the OFC. This result is consistent with the previous studies mostly using anterograde tracers; neurons in the OFC mainly innervate the rostral part of striatum (Berendse et al., 1992; Schilman et al., 2008; Mailly et al., 2013). It still remains a question whether this small number of TS-projecting neurons have a role in value processing.

### Cortical Layer Distribution of TS- and DMS-Projecting Neurons

We found that both layer 5 and 6 in some cortical regions projected to the TS. The cortical layer 5 is known to be a major input to the striatum, but some studies have shown that the cortical layer 6 also innervates the primate CDt and the rodent striatum (Gerfen, 1989; Saint-Cyr et al., 1990; Hintiryan et al., 2016; Griggs et al., 2017) These different layer inputs to the TS and the DMS might give an idea of the functional differences to guide habitual and goal-directed behavior. It is possible that some of the retrograde labeling occurred via damaged axon en passage. However, our data showed that the TS selectively received inputs from layer 5 and 6 in the cortex whereas the DMS mostly received inputs from the layer 5 (an example of the parietal association cortex in Figure 8B). Furthermore, we did not find the retrogradely labeled neurons in layer 6 of the parietal association cortex when the tracers were injected above the TS, mainly in the external capsule (from one rat; data not shown). It is unlikely that such erroneous labeling strongly affected the results.

### Sensory Input Difference Between Primate CDt and Rat TS

The caudate tail of macaque monkeys mainly receives sensory inputs from the visual cortex and no or weak inputs from the somatosensory and olfactory regions (Saint-Cyr et al., 1990; Yeterian and Pandya, 1995; Griggs et al., 2017). In contrast, our rat data and previous mouse studies show that the TS receives inputs from the various sensory cortices including the visual cortex (Hintiryan et al., 2016; Hunnicutt et al., 2016). One general difference between the rodent and primate striatum is structural segregation. For example, the caudate and putamen are separated in primates but structurally merged in rodents. The TS in rodents might be merged with some parts of the primate caudate and putamen. Thus, we found that all sensory inputs were merged in the TS. This result suggests that all sensory-response associations may be processed in the rat TS, but the primate striatum is functionally more segmented.

### Technical Consideration

As a general consideration in all retrograde tracer studies, some of the retrograde labeling may occur via damaged axons of passage near the injection site. In our tracer study, it is unlikely that this erroneous labeling heavily affected our results for two reasons. First, five rats had similar results, which is unlikely if there was substantial uptake by axons of passage. Second, all of the injection sites had minimal leakage. However, we still cannot exclude the possibility of en passage labeling completely. Addressing this issue will require further experiments, such as anatomical and functional tracings with genetic methods in future.

### Future Question: What Are the Different Roles of the DLS and TS in Habitual Behavior?

One interesting question remains because the DLS is also known to guide habitual behavior; what are the anatomical and functional differences and similarities between the TS and the DLS? Based on previous reports (Fuccillo, 2016; Hintiryan et al., 2016), the brain regions innervating the TS seem to be very similar to those innervating the DLS. Together with our anatomical data, identifying what are the similar and different inputs to the TS, DMS and DLS would be important to understand their distinct roles and functional relationships in habitual and goal-directed behavior.

## Author Contributions

HJ and HFK designed the research, performed the research and wrote the article. HJ analyzed the data with input from HFK.

## Conflict of Interest Statement

The authors declare that the research was conducted in the absence of any commercial or financial relationships that could be construed as a potential conflict of interest.
