# Supplementary material for: Anatomical Inputs From the Sensory and Value Structures to the Tail of the Rat Striatum
Source: Front Neuroanat. 2018 May 3;12:30. doi: 10.3389/fnana.2018.00030 (PMC5943565; doi:10.3389/fnana.2018.00030)
Supplement: Supplementary file 1 [file Image_1.pdf]

A

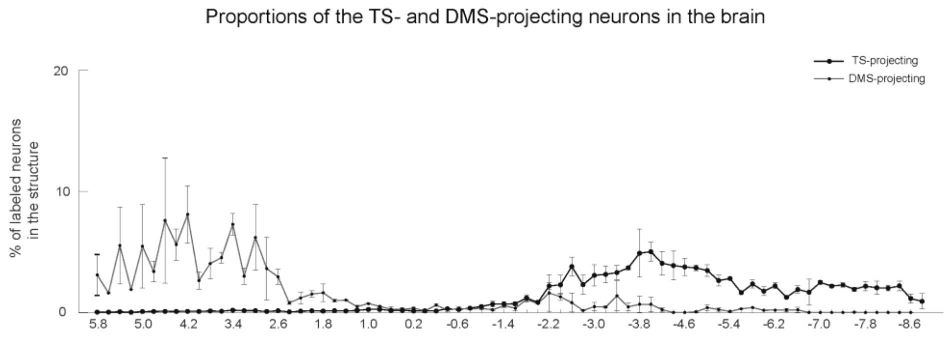

Supplementary figure 1. Topographic projections to the TS and DMS from the rostral-caudal axis. (A) Distributions of TS- (black line) and DMS-projecting (grey line) neurons at 200-µm intervals in the rostral-caudal axis (TS-projecting neurons, n = 3 hemispheres; DMS-projecting neurons, n = 2 hemispheres).

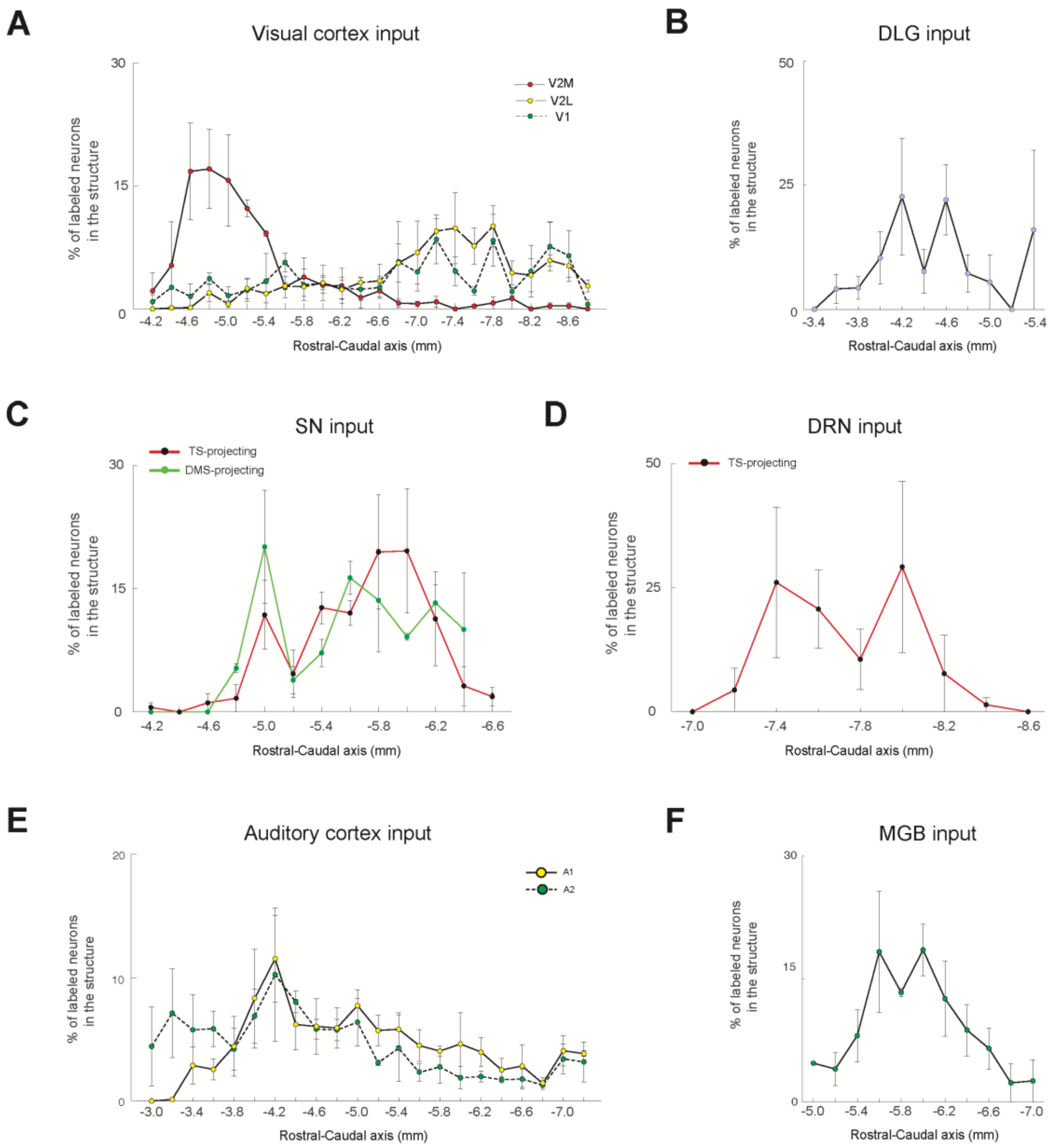

Supplementary figure 2. Sensory and value inputs to the TS and DMS. (A) Rostral-caudal distribution of TS-projecting neurons at 200-μm intervals in the subregions of the visual cortex (n = 3 hemispheres). V1, primary visual cortex; V2L, lateral part of the secondary visual cortex; V2M, medial part of the secondary visual cortex. **(B)** Rostral-caudal distribution of TS-projecting neurons at 200-μm intervals in the DLG (n = 3 hemispheres). DLG, dorsolateral geniculate nucleus. **(C)** Rostral-caudal distributions of TS- and DMS-projecting neurons at 200-μm intervals in the SN (n = 3 hemispheres and n = 2 hemispheres, respectively). SN, substantia nigra. **(D)** Rostral-caudal distribution of TS-projecting neurons at 200-μm intervals in the DRN (n = 3 hemispheres). DRN, dorsal raphe nucleus. **(E)** Rostral-caudal distribution of TS-projecting neurons at 200-μm intervals in the subregions of the auditory cortex (n = 3 hemispheres). A1, primary auditory cortex; A2, secondary auditory cortex. **(F)** Rostral-caudal distribution of TS-projecting neurons at 200-μm intervals in the MGB (n = 3 hemispheres). MGB, medial geniculate body.

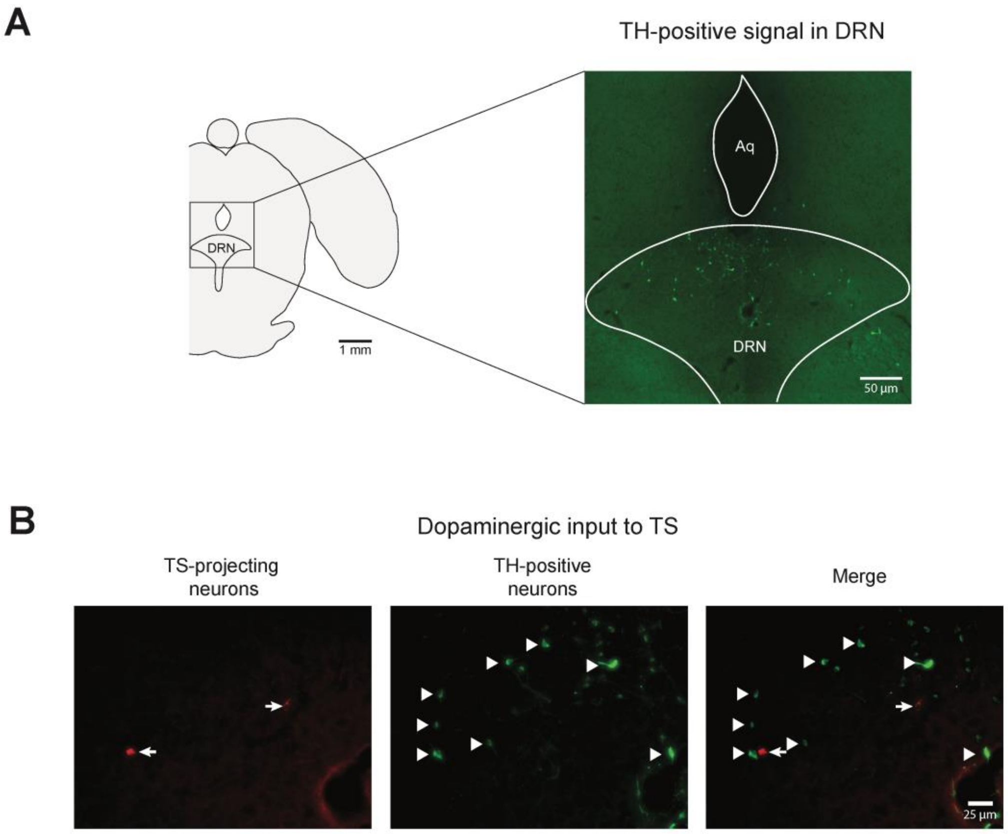

Supplementary figure 3. TS-projecting neurons and TH-positive dopamine neurons in the dorsal raphe nucleus. (A) Tyrosine hydroxylase (TH)-positive neurons (green signal) were found in the dorsal raphe nucleus (DRN). **(B)** TS-projecting neurons in the DRN were not colocalized with TH-positive dopamine neurons.

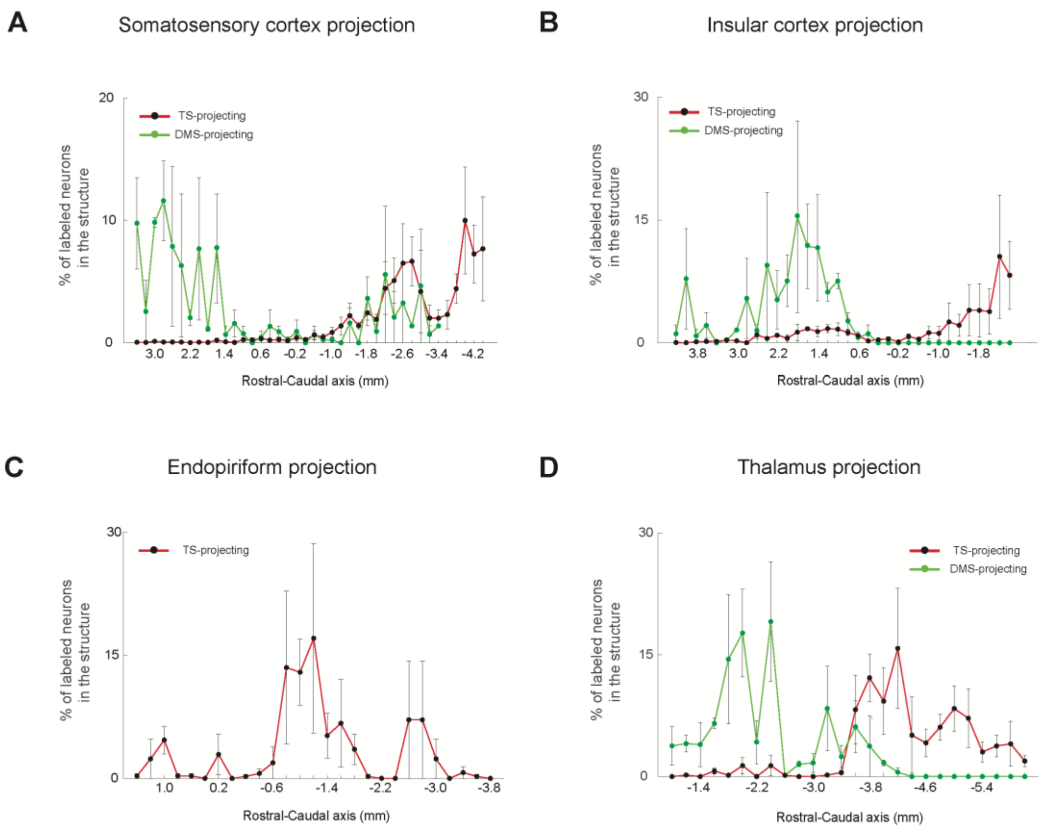

Supplementary figure 4. Inputs from the somatosensory, insular, endopiriform cortex and the thalamus to the TS and DMS. (A) Rostral-caudal distribution of TS- (red line) and DMS-projecting (green line) neurons at 200-µm intervals in the somatosensory cortex (TS-projecting neurons, n = 3 hemispheres; DMS-projecting neurons, n = 2 hemispheres). **(B)** The same format as in (A), showing projections from the insular cortex. **(C)** The same format as in (A), showing inputs from the endopiriform cortex. **(D)** The same format as in (A), showing projections from the thalamus.

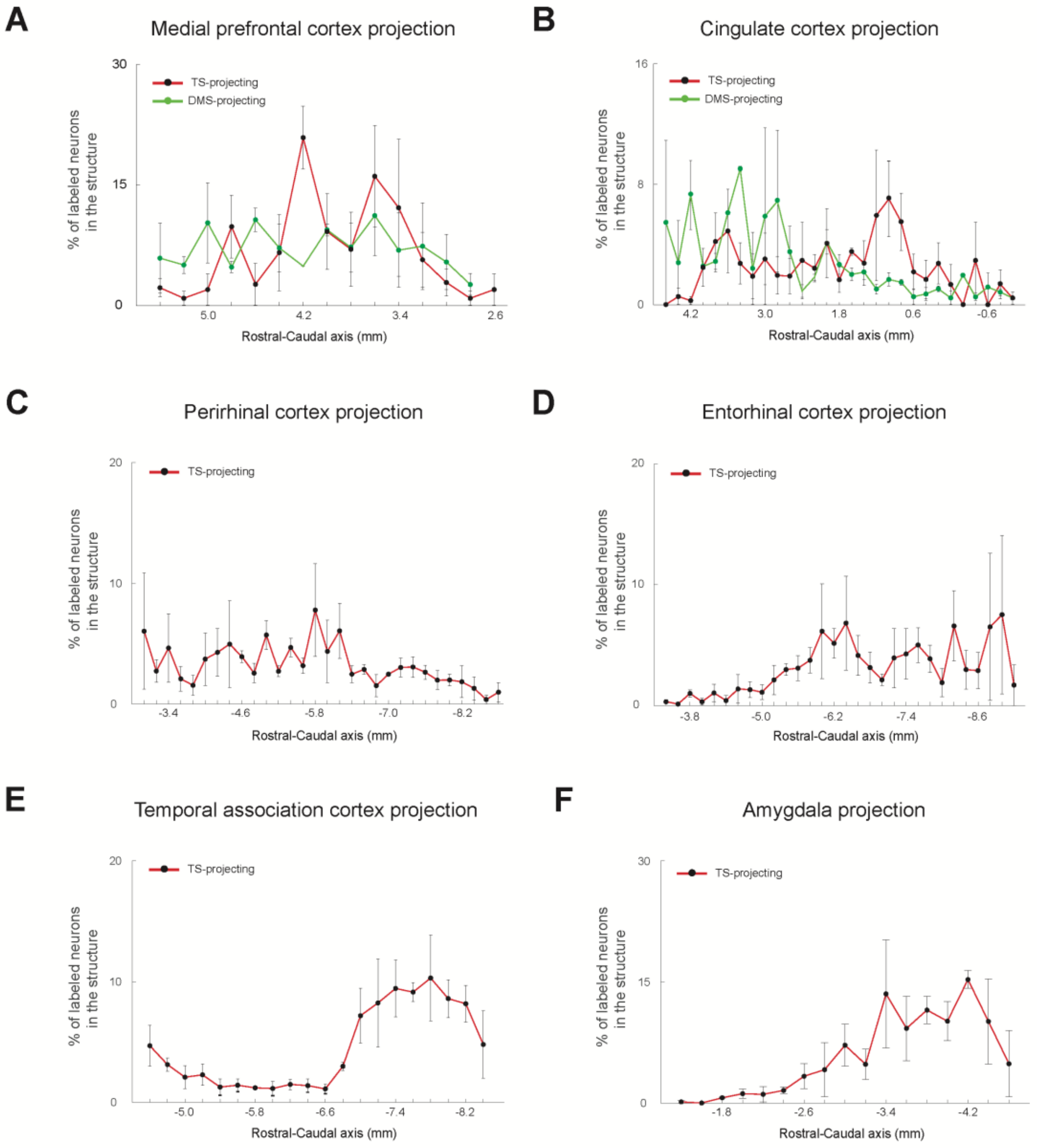

Supplementary figure 5. Projections from the limbic structures to the TS and DMS. **(A)** Rostral-caudal distribution of TS- (red line) and DMS-projecting (green line) neurons at 200-μm intervals in the medial prefrontal cortex (TS-projecting neurons, n = 3 hemispheres; DMS-projecting neurons, n = 2 hemispheres). **(B)** The same format as in (A), showing inputs from the cingulate cortex. **(C)** The same format as in (A), showing inputs from the perirhinal cortex. **(D)** The same format as in (A), showing inputs from the entorhinal cortex. **(E)** The same format as in (A), showing inputs from the temporal association cortex. **(F)** The same format as in (A), showing inputs from the amygdala.

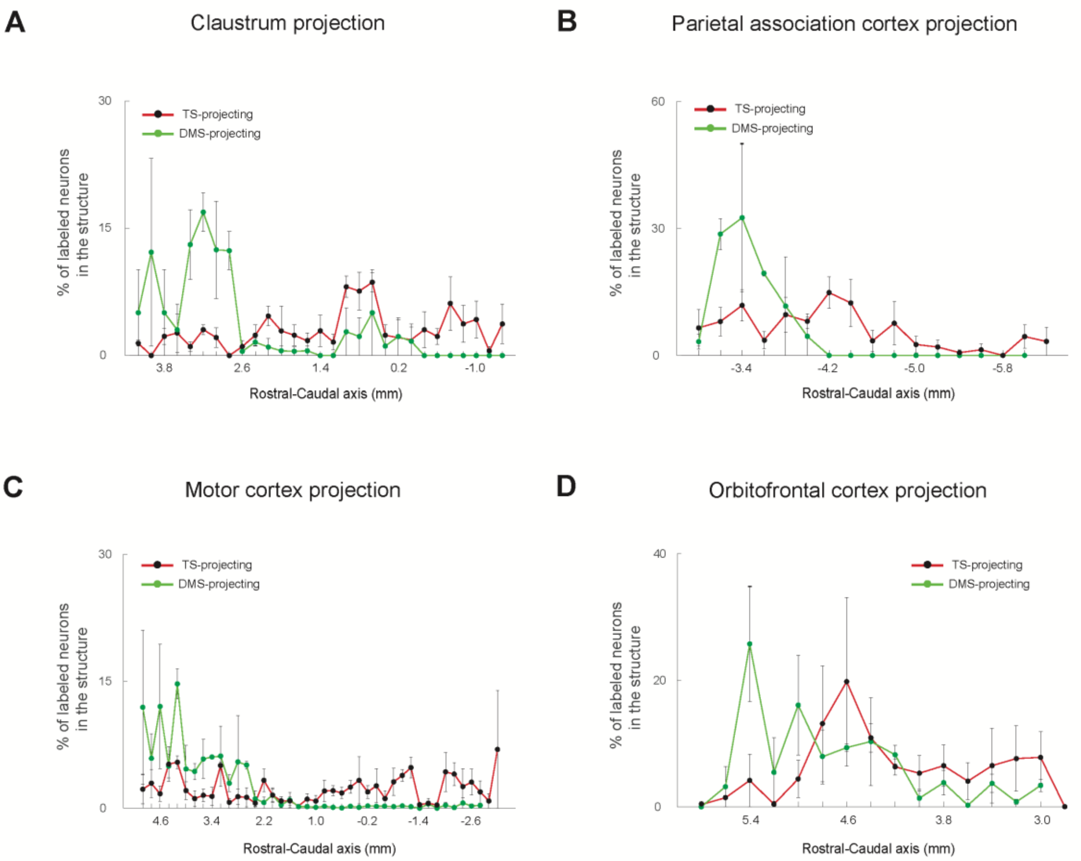

Supplementary figure 6. Inputs from the sensory associative structures, motor cortex, and orbitofrontal cortex to the TS and DMS. (A) Rostral-caudal distribution of TS- (red line) and DMS-projecting (green line) neurons at 200-µm intervals in the claustrum (TS-projecting neurons, n = 3 hemispheres; DMS-projecting neurons, n = 2 hemispheres). **(B)** The same format as in (A), showing inputs from the parietal association cortex. **(C)** The same format as in (A), showing inputs from the motor cortex. **(D)** The same format as in (A), showing inputs from the orbitofrontal cortex.
